# Application and Development of Spectral CT in Target Delineation for Lung Cancer Radiotherapy

**DOI:** 10.3390/diagnostics16172859

**Published:** 2026-09-05

**Authors:** Shanshan Zhang, Bijing Mao

**Affiliations:** 1 Department of Radiology, The Third Affiliated Hospital of Chongqing Medical University (Fangda Hospital), Chongqing 401120, China; 651790@hospital.cqmu.edu.cn; 2 Department of Oncology, The Third Affiliated Hospital of Chongqing Medical University (Fangda Hospital), Chongqing 401120, China

**Keywords:** spectral CT, lung cancer, radiotherapy, target volume delineation, material decomposition, iodine map

## Abstract

Lung cancer ranks first among all malignancies in both incidence and mortality worldwide, and precise target volume delineation is the key to ensuring the efficacy and safety of radiotherapy. Conventional CT imaging has significant limitations in distinguishing tumors from surrounding normal tissues, particularly in the presence of atelectasis or inflammation, which compromises the accuracy of target delineation. As an advanced imaging technology, spectral CT can provide multi-dimensional information, including material composition analysis, monoenergetic images, and effective atomic number mapping, offering groundbreaking potential to enhance the precision of lung cancer target volume delineation. This article provides a comprehensive overview of the fundamental principles of spectral CT, explores its specific application value in lung cancer target volume delineation, analyzes the current challenges, and provides an outlook on future development trends, aiming to serve as a comprehensive reference for clinical practice and scientific research.

## 1. Introduction

Lung cancer ranks among the malignancies with the highest incidence and mortality rates globally, and its radiotherapy is highly dependent on precise imaging evaluation [1]. Radiotherapy is an important treatment modality for locally advanced non-small cell lung cancer (NSCLC), and its efficacy and toxicity are directly determined by the accurate definition of tumor targets [2]. Conventional computed tomography (CT) serves as the cornerstone of radiotherapy planning, primarily relying on the linear attenuation coefficient (CT number) of tissues for imaging. However, when tumors exhibit densities similar to adjacent atelectasis, inflammation, or necrosis, conventional CT suffers from insufficient contrast and specificity, which may lead to target delineation that is either too large—increasing irradiation doses and complication risks to normal tissues (such as lung, heart, and spinal cord)—or too small, resulting in target miss and local recurrence [3,4]. For instance, a study by Li et al. demonstrated that while conventional venous-phase CT could only distinguish central tumors from atelectasis in 33.33% of cases, 40 keV monoenergetic images, and iodine density maps achieved differentiation rates of 68.63% and 76.47%, respectively [5]. Furthermore, conventional CT has inherent limitations in evaluating lymph node metastasis, intratumoral heterogeneity, and predicting molecular marker status [4,6].

To address these challenges, spectral CT technology has emerged and continues to evolve. This technology synchronously acquires attenuation data at both high and low energy levels using dual-energy X-ray sources or rapid kilovoltage switching [6]. Through material decomposition algorithms, it generates various functional images, such as monoenergetic images, material basis images, and spectral curves [6,7]. Monoenergetic images, particularly at low kiloelectron volt (keV) levels (e.g., 40 keV), significantly enhance the contrast of iodine-containing tissues, facilitating the detection of occult lesions. Iodine density maps can directly quantify tissue iodine uptake, reflecting blood supply status and providing objective evidence for differentiating viable tumors from hypovascular areas (necrosis, atelectasis) [4,5,7]. Studies have confirmed that venous-phase spectral CT is significantly superior to conventional CT in distinguishing central lung cancer from atelectasis [5]. Additionally, quantitative parameters of spectral CT (such as spectral curve slope) are associated with tumor biological behaviors (e.g., Ki-67 expression, EGFR mutation status), opening new avenues for non-invasive prediction of tumor aggressiveness and therapeutic targets [6,8]. However, it is important to note that these findings are primarily exploratory, and the correlation between spectral parameters and molecular biomarkers requires broader validation across independent cohorts using standardized acquisition protocols before it can be translated into routine clinical practice.

In recent years, with the continuous advancement of spectral CT hardware and image post-processing techniques, research on its application in precision radiotherapy for tumors has deepened. Beyond optimizing initial target delineation, spectral CT has also shown broad prospects in evaluating radiotherapy response, guiding adaptive radiotherapy, and integrating with artificial intelligence (AI) [6,8]. The functional fusion of spectral CT with positron emission tomography (PET) enables the simultaneous evaluation of tumor glucose metabolism activity and iodine-enhanced blood supply, providing a more comprehensive basis for target delineation and response assessment [7]. Currently, ^18^F-FDG PET/CT has demonstrated clear value in adjusting radiotherapy plans (e.g., more accurate identification of lymph node and distant metastases, thereby altering staging and treatment strategies). As a more accessible contrast-enhanced CT technique with quantitative capabilities, spectral CT is expected to play a unique role in distinguishing malignant from benign lesions and reducing inter-observer variability in delineation [5,9]. This article will focus on the technical principles and quantitative advantages of spectral CT, and summarizes the current evidence regarding its application status, clinical evidence, challenges, and future development trends in various aspects of lung cancer target volume delineation, including primary tumor definition, lymph node assessment, response monitoring, and adaptive radiotherapy. However, despite this progress, there remain substantial gaps between technological promise and routine clinical implementation. These gaps encompass a lack of technical standardization, limited evidence from large-scale prospective trials, challenges in clinical workflow integration, and the associated cost-effectiveness uncertainties, all of which will be systematically addressed in the following sections.

Scope of this review: This review aims to provide a comprehensive overview of the current clinical applications, technical principles, available evidence, and future directions of spectral CT technology specifically in the context of target delineation for lung cancer radiotherapy. We focus on the unique advantages of spectral CT parameters—including iodine density maps, effective atomic number (Z-effective) maps, and monoenergetic images—in addressing the specific challenges of lung cancer target volume definition, such as distinguishing tumors from adjacent inflammatory atelectasis and improving the precision of gross tumor volume (GTV) delineation. The integration of spectral CT with other multimodal imaging modalities and artificial intelligence is discussed with particular emphasis on their relevance to lung cancer radiotherapy. By synthesizing the existing evidence and identifying current knowledge gaps, this review seeks to provide a practical reference for radiation oncologists and to inform future research directions in this evolving field.

To achieve the objectives outlined above, this review begins with the technical principles and imaging characteristics of spectral CT, elucidating the core advantages that distinguish it from conventional CT—this serves as the prerequisite and foundation for understanding all subsequent clinical applications.

While the subsequent sections will elaborate on these promising applications, it is equally important to recognize that the clinical translation of spectral CT faces substantial barriers. These include the lack of cross-vendor quantitative standardization, the absence of radiotherapy-specific validated thresholds, limited prospective outcome data, and unresolved cost-effectiveness concerns. A balanced appraisal of both opportunities and limitations is therefore essential before spectral CT can be recommended for routine target delineation in lung cancer radiotherapy.

Based on the evidence synthesized in this review, we propose the following stepwise cognitive framework for integrating spectral CT into lung cancer target delineation: (i) patient selection—prioritize spectral CT for central lung tumors with ambiguous boundaries on conventional CT due to obstructive atelectasis or inflammation; (ii) primary interpretation—utilize venous-phase iodine density maps as the principal reference for identifying viable tumor (high iodine uptake) versus necrosis or atelectasis (low uptake); (iii) confirmatory cross-validation—employ low-keV (40–50 keV) virtual monoenergetic images and effective atomic number maps to refine boundary definition; (iv) treatment planning integration—use virtual non-contrast images derived from the same acquisition for dose calculation; (v) dynamic monitoring—track longitudinal changes in iodine concentration during radiotherapy as an exploratory biomarker for adaptive response assessment. This framework provides a practical clinical roadmap while acknowledging the need for future quantitative refinement through prospective studies.

## 2. Technical Principles and Imaging Characteristics of Spectral CT

The core distinction of spectral CT from conventional CT lies in its multi-parameter imaging capability. Understanding these technical principles—particularly the generation mechanisms of iodine density maps, effective atomic number (Z-effective) maps, and monoenergetic images—is the first step in evaluating its application value in lung cancer radiotherapy target delineation.

### 2.1. Dual-Energy Data Acquisition and Material Decomposition Principles

The core of spectral CT imaging lies in the energy-dependent attenuation characteristics of X-rays interacting with matter. By acquiring attenuation data at different energies, spectral CT overcomes the limitations of conventional CT in tissue characterization [10]. Three main technical approaches exist for achieving dual-energy acquisition: dual-source CT (two tube–detector combinations, with one tube emitting low-energy rays and the other emitting high-energy rays, scanning simultaneously at the same angle), rapid kilovoltage switching with a single source (a single tube alternates between high and low voltages in an extremely short time, achieving near-simultaneous data acquisition), and dual-layer detector technology (detector-end stacking design for simultaneous acquisition of high- and low-energy data) [11,12,13]. The physical basis of material decomposition lies in the differential energy dependence of the photoelectric effect and Compton scattering for different materials [12]. Through mathematical models, the attenuation information of the scanned object can be decomposed into two or three basis material pairs (e.g., iodine–water, calcium–water), enabling the quantitative determination of specific substances (e.g., iodine) concentrations [11]. This process transcends the limitation of conventional CT, which only provides a single CT number, and can generate quantitative information reflecting tissue chemical composition, thereby laying a solid physical foundation for accurately distinguishing different tissue types [10]. Recently, the advent of photon-counting detector CT marks a technological leap, as it can directly count the energy of each photon, achieving inherent and higher-precision spectral imaging [14,15].

### 2.2. Spectral CT-Derived Image Types and Their Clinical Significance

Monoenergetic Images: Through mathematical algorithms, these images simulate the effect of imaging at any single energy level from 40 to 200 keV. Low-keV images (e.g., 40–50 keV) greatly improve the contrast-to-noise ratio (CNR) of iodine-containing tissues. This feature significantly enhances the visualization of tumor neovascularization and actively enhancing areas, making lesion margins, internal necrotic zones, and infiltration ranges more visually prominent. This is particularly helpful for clearly delineating lesion boundaries when adjacent tissues have similar densities, reducing subjective interpretation errors [13]. Conversely, high-keV images (e.g., 100–140 keV) can effectively suppress beam-hardening and metal artifacts caused by high-density materials (such as bone, metal implants, and high-concentration contrast agents), restoring the true density information of surrounding soft tissues and providing a more reliable anatomical background for target delineation and dose calculation [13].

Material Decomposition Images: Through complex material separation algorithms, spectral CT decomposes voxels within the scan volume into combinations of two or three basis material pairs (e.g., iodine–water, calcium–water). Among these, the iodine map enables visualization and absolute concentration quantification (unit: mg/mL) of iodine distribution within tissues, assessing tumor blood supply and perfusion, and providing a basis for determining tumor activity. This offers an objective imaging biomarker for distinguishing highly vascular viable tumor tissue from hypovascular necrosis, fibrosis, or inflammatory lesions, demonstrating unique value particularly in differentiating central lung cancer from obstructive atelectasis [11]. In addition, virtual non-contrast (VNC) images are synthesized by precisely removing the iodine signal from contrast-enhanced scans through intelligent algorithms, producing images equivalent to true non-contrast scans. This approach reduces radiation dose by approximately 30–50% by eliminating the need for a separate non-contrast acquisition, as documented across multiple clinical studies [16,17]. However, while VNC images are acceptable for many clinical tasks, they are not a perfect substitute for true non-contrast (TNC) in all scenarios—equivalence varies by tissue type and acquisition phase, with attenuation values for certain organs (e.g., liver, spleen, and pancreas) showing significant differences compared with true non-contrast images [18]. For radiotherapy planning specifically, VNC has demonstrated dosimetric equivalence to TNC for dose calculation purposes, with reported differences of less than 0.2% in planning target volume (PTV) mean and maximum doses [19]; however, its use for target delineation and quantitative assessment should be verified in the specific clinical context, particularly for small structures and subtle lesions.

Spectral Curves and Quantitative Parameters: Spectral curves (or attenuation curves) depict the continuous variation trajectory of tissue CT values from low keV to high keV within a region of interest, constructing a unique “energy fingerprint” for that tissue. Because tissues with different chemical compositions (e.g., tumor parenchyma, necrosis, inflammatory tissue, lymph nodes) exhibit different relative contributions of the photoelectric effect and Compton scattering with energy variation, their spectral curve morphologies (e.g., slope, curvature) also differ distinctly [20]. This feature provides a new dimension beyond traditional CT numbers for qualitative tumor diagnosis and homology analysis—for example, quantitatively assessing iodine uptake capacity via curve slope, or differentiating metastatic from reactive hyperplastic lymph nodes. Meanwhile, the effective atomic number map (Z_eff_) can calculate and display the effective atomic number of each voxel, a parameter that reflects the spectral characteristics of tissue X-ray attenuation and can be used to indirectly characterize the predominant elemental composition and biological properties of tissues. Clinically, effective atomic number helps differentiate tissues with similar compositions but different properties (e.g., calcification vs. iodine accumulation, uric acid stones vs. calcium-containing stones) and serves as a potential imaging indicator for assessing tumor heterogeneity or microenvironmental changes, assisting in more precise differential diagnosis and biological behavior inference [21,22].

These quantitative image types—including monoenergetic images, iodine maps, and effective atomic number maps—are generated through mathematical transformations of spectral raw data. In principle, their theoretical foundation (e.g., the energy-dependent attenuation behavior of materials) is independent of scanning conditions such as tube voltage and patient body size, which distinguishes them from conventional CT numbers and provides a conceptual basis for standardized multi-center applications [23].

However, it is crucial to recognize that this theoretical advantage does not automatically translate into practical cross-device reproducibility. Currently, different vendors employ fundamentally distinct hardware architectures to achieve spectral imaging—for example, GE’s rapid kVp switching, Siemens’ dual-source configuration, and Philips’ dual-layer detector design. These proprietary differences extend to raw data processing and material decomposition algorithms, often yielding quantitatively distinct results (e.g., iodine concentration values) for the same tissue when measured across different platforms. Consequently, there remains a notable lack of universally accepted reference standards for key quantitative parameters, which substantially hinders the direct cross-comparison of results from different centers or devices and limits the advancement of large-scale multi-center validation studies [23]. Moreover, even on the same device, scan protocol variations—including tube voltage selection, contrast agent injection regimens, and acquisition phase timing—can significantly alter the final quantitative outputs, further compounding the reproducibility challenge.

Therefore, while spectral CT offers unprecedented biological characterization capabilities that move precision radiotherapy beyond “morphological delineation” toward “biological characterization” [23], the realization of this potential depends critically on overcoming the current barriers of technical standardization and cross-vendor harmonization—a topic that will be systematically addressed in Section 5.1. For radiation oncologists, this means that despite the impressive theoretical capabilities, spectral CT-derived parameters should currently be interpreted with caution and used as adjunctive information rather than standalone decision-making criteria in target delineation.

In summary, the multi-parameter framework of spectral CT theoretically possesses the technical advantages of differentiating tissue components and quantifying tissue characteristics. Whether these features can translate into tangible improvements in delineation accuracy within the clinical practice of lung cancer target delineation will be systematically reviewed in the following section.

## 3. Specific Application Value of Spectral CT in Lung Cancer Target Volume Delineation

Building upon the technical principles discussed above, this section focuses on the clinical evidence for individual spectral CT quantitative parameters in specific steps of lung cancer radiotherapy target delineation. We focus on the practical value of iodine density maps, Z-effective maps, and monoenergetic images in distinguishing lung tumors from adjacent inflammatory atelectasis, defining GTV boundaries, and optimizing target volume precision.

### 3.1. Improving Differentiation Between Tumor and Atelectasis/Inflammation

**Figure 1:**
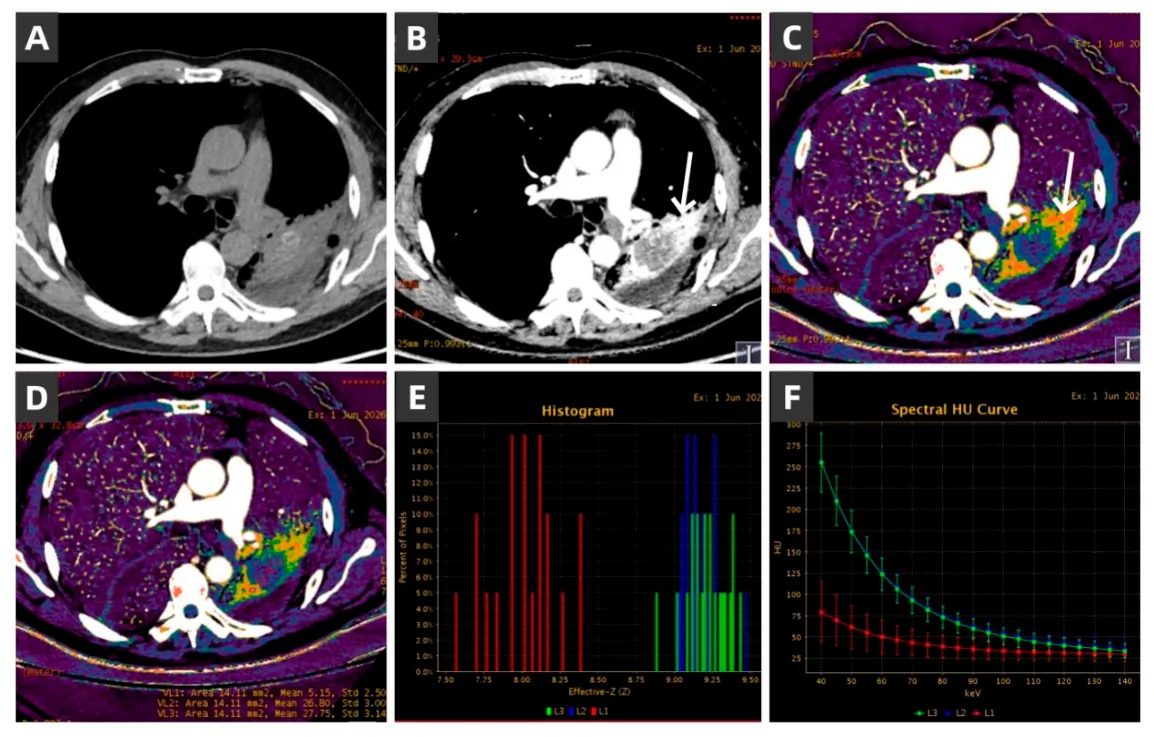
Spectral CT multi-parametric imaging for differentiating tumors from atelectasis or necrosis. (**A**) Conventional contrast-enhanced CT image (120 kVp) shows that the tumor and adjacent atelectatic lung tissue exhibit similar attenuation, with an ill-defined boundary. (**B**) 40 keV virtual monoenergetic image (VMI) demonstrates that the hypervascular tumor shows marked hyperenhancement (white arrows), whereas the atelectatic region exhibits low enhancement, allowing clear delineation of the boundary. (**C**) Iodine density map with color coding: warm colors (red/yellow) represent high iodine concentration, indicating viable tumor regions (white arrows); cold colors (blue/green) represent low iodine concentration, indicating necrosis or atelectasis. (**D**) Regions of interest (ROIs) are placed on the iodine density map in the atelectasis or necrosis (L1) and two different tumor portions (L2, L3). (**E**) Effective atomic number (Z_eff_) values for each ROI, showing that the Z_eff_ value of the atelectasis or necrosis region (red) is significantly lower than that of the tumor regions (green and blue). (**F**) Spectral attenuation curves of the three ROIs over the 40–140 keV range, demonstrating marked differences in curve morphology between the tumor regions (green and blue) and the atelectasis or necrosis region (red).

Central lung cancer is often accompanied by obstructive atelectasis or pneumonia. On conventional CT images, the tumor and atelectatic lung tissue have blurred boundaries due to similar densities, creating significant difficulties for accurate tumor target delineation. Spectral CT, by providing material decomposition images—particularly iodine maps—can clearly distinguish the highly vascular tumor (high iodine concentration) from the hypovascular atelectasis/inflammation (low iodine concentration) [5,24]. A study involving 51 patients showed that conventional CT in the venous phase could only distinguish tumor from atelectasis in 33.33% of cases, whereas 40 keV monoenergetic images, iodine maps, and their fused images achieved differentiation rates of 68.63%, 76.47%, and 74.51%, respectively [5]. This study indicates that the GTV delineated based on iodine maps or low-keV monoenergetic images differs significantly from that based on conventional CT, typically being smaller in volume and more accurate in boundary definition, thereby potentially avoiding excessive irradiation of atelectatic lung tissue and reducing the risk of radiation-induced normal lung tissue damage while improving tumor control probability [5]. Beyond iodine maps, effective atomic number maps can also provide useful Supplementary Information. Research indicates that the effective atomic number of tumor tissue is typically higher than that of benign atelectatic tissue, offering another quantitative indicator for differentiation [25]. For example, significant differences in effective atomic number exist between invasive thymic epithelial tumors and mediastinal lung cancer [25]. Additional studies suggest that spectral CT has the potential to replace pulmonary perfusion CT for evaluating tumor histological properties and hemodynamics [26]. Thus, through multi-parametric quantitative analysis, spectral CT significantly enhances the ability to identify the true boundaries of central lung cancer, laying a solid foundation for precision radiotherapy (Figure 1).

Nevertheless, several caveats warrant attention. First, the reported differentiation rates (e.g., 76.47% on iodine maps) indicate that a substantial proportion of cases still exhibit ambiguous boundaries, suggesting that spectral CT does not completely resolve the tumor–atelectasis distinction in all patients. Second, the optimal imaging phase (arterial vs. venous) for target delineation remains debated, and the dynamic changes in iodine uptake during the respiratory cycle have not been systematically studied. Third, the utility of these parameters may be compromised in patients with poor cardiac output or suboptimal contrast enhancement. Therefore, while spectral CT offers meaningful improvement, it should be interpreted as a complementary aid rather than a definitive solution.

### 3.2. Optimizing the Delineation of Gross Tumor Volume (GTV) and Clinical Target Volume (CTV)

**Figure 2:**
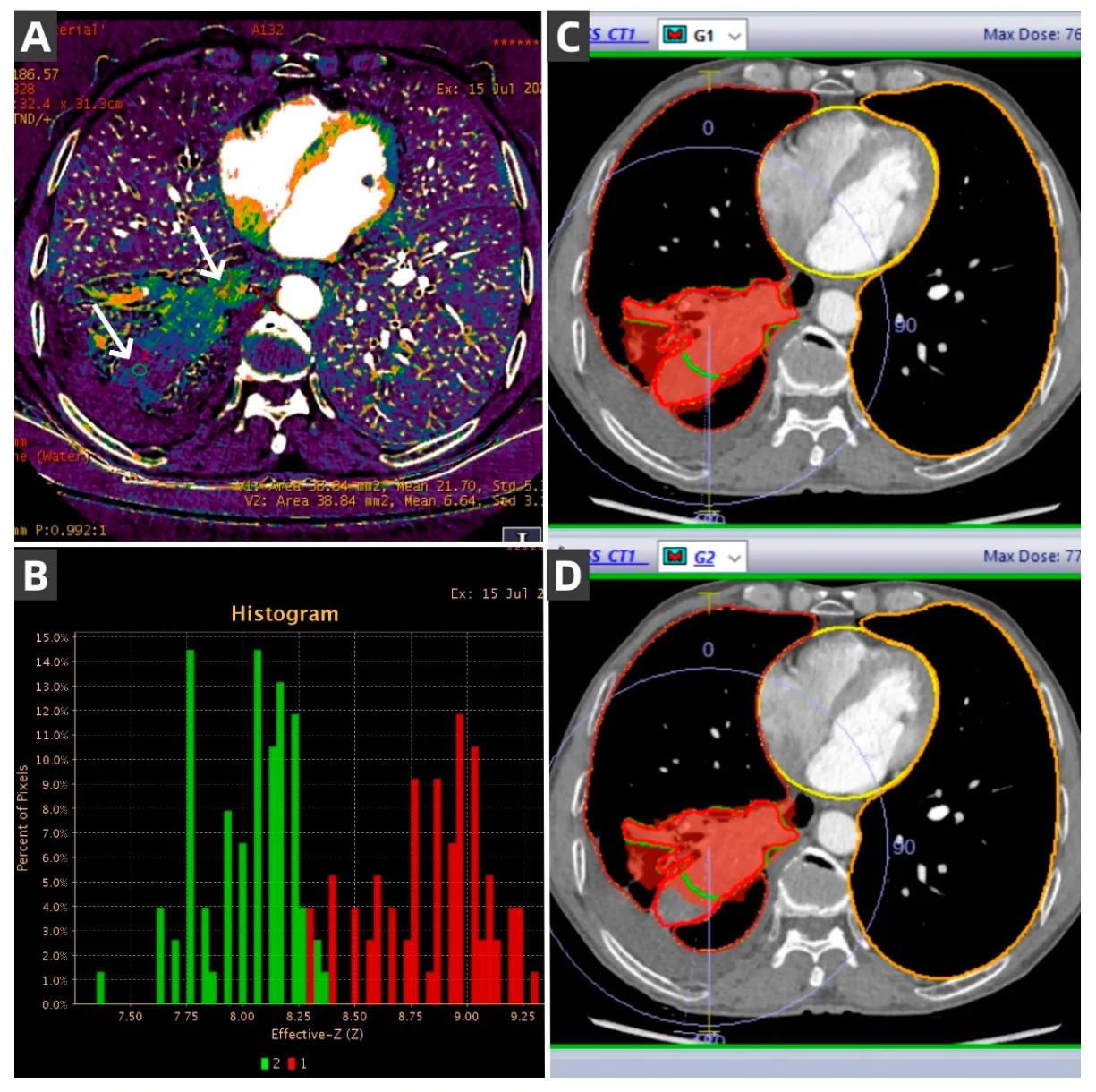
Iodine map-based delineation of radiotherapy target volume reduces inclusion of necrotic or atelectatic regions compared with conventional CT. (**A**) Two regions of interest (ROIs) are delineated on the iodine density map (white arrows): ROI 1 (red) shows high iodine concentration, indicating viable tumor tissue; ROI 2 (green) shows low iodine concentration, indicating necrosis or atelectasis. (**B**) Effective atomic number (Z_eff_) values corresponding to the two ROIs, demonstrating that the Z_eff_ value of the necrosis or atelectasis region (green) is significantly lower than that of the tumor region (red). (**C**) Radiotherapy target volume (red shaded area) delineated based on conventional contrast-enhanced CT. (**D**) Iodine map-based target volume delineation (red shaded area), which excludes necrosis or atelectatic regions and results in a substantially smaller volume, thereby potentially avoiding unnecessary irradiation of non-viable atelectatic lung tissue.

Spectral CT not only clearly defines the external contours of tumors but also reveals their internal heterogeneity. Through iodine concentration analysis, spectral CT can differentiate necrotic areas (low iodine) from viable areas (high iodine) within the tumor [27]. Such functional imaging information enables further delineation of biological target volumes within the GTV—for example, implementing dose escalation (boost) targeting areas with high iodine uptake, providing imaging evidence for dose painting, a precision radiotherapy technique [27]. It is essential to emphasize, however, that this remains an exploratory concept and a subject of active research. The translation of intratumoral iodine heterogeneity into a safe and effective dose painting strategy requires prospective validation in clinical trials that correlate the distribution of dose with local tumor control and toxicity outcomes. When determining the CTV (which includes the GTV and its subclinical infiltration range), spectral parameters assist in more accurately assessing the microscopic invasive tendency of tumors. For instance, spectral CT parameters correlate with Ki-67 expression levels reflecting tumor proliferative activity, with significant differences in venous-phase normalized iodine concentration and spectral curve slope between high and low Ki-67 expression groups [28]. Although this area remains a current research hotspot, it has already demonstrated the potential for non-invasive prediction of tumor biological behavior through imaging. Furthermore, by more clearly displaying the relationship between tumors and adjacent critical structures (such as vessels and bronchi), spectral CT helps reduce misjudgments regarding whether these structures are invaded during target delineation. For example, spectral CT demonstrates high efficacy in distinguishing non-small cell lung cancer (NSCLC) from small-cell lung cancer (SCLC), with venous-phase normalized iodine concentration showing the highest diagnostic performance [29]. This accurate pathological subtyping, combined with the clear depiction of tumor relationships with surrounding structures, holds significant reference value for individualizing CTV delineation (e.g., SCLC typically requires more extensive prophylactic irradiation fields) (Figure 2).

Importantly, the proposed use of spectral CT parameters for biological target volume delineation and dose painting remains largely conceptual. The correlation between iodine uptake and Ki-67 expression, while statistically significant in some studies, does not automatically translate into a safe or effective dose-escalation strategy. Prospective clinical trials correlating spectral CT-guided dose distribution with local control and toxicity outcomes are urgently needed before such approaches can be adopted in routine practice.

### 3.3. Aiding Lymph Node Staging and Target Volume Delineation

Accurate lymph node staging is crucial for treatment decision-making and prognosis evaluation in lung cancer. Conventional CT primarily relies on lymph node size for assessment, with limited specificity. Quantitative analysis via spectral CT, particularly lymph node iodine uptake quantification on iodine density maps, may offer higher specificity than size measurement alone [30]. Studies have shown that multiple quantitative parameters from dual-source spectral CT (e.g., arterial-phase 40 keV normalized attenuation rate, normalized iodine concentration, etc.) differ significantly between metastatic and non-metastatic groups when predicting lymph node metastasis in NSCLC [30]. For radiotherapy target volumes that need to encompass lymph node regions, spectral CT provides more accurate morphological and functional information. For example, in diagnosing N2 station lymph node metastasis in T1-stage NSCLC, metastatic lymph nodes exhibited lower values on 40 keV monoenergetic CT numbers, spectral curve slope, normalized iodine concentration, and normalized effective atomic number compared to non-metastatic lymph nodes [31]. Combining venous-phase spectral quantitative parameters with lymph node short-axis diameter significantly improves diagnostic efficacy, facilitating individualized determination of lymph node CTV extent and avoiding over-irradiation of negative lymph node regions or under-dosing of positive ones [31]. The functional information provided by spectral CT can assist in judging lymph node properties, though its parameters do not show correlation with PET/CT parameters in tumors and metastatic lymph nodes, suggesting they reflect different tumor characteristics and should complement rather than replace each other [32]. This is a crucial point, as it indicates that spectral CT may provide incremental diagnostic benefit, particularly in settings where PET/CT is unavailable or contraindicated, or in clarifying ambiguous findings. However, it should be noted that while spectral CT provides functional information similar to PET/CT, its ability to detect distant metastases is inferior to that of whole-body PET/CT. The incremental value of spectral CT is therefore best realized in the detailed local assessment of lymph nodes at specific stations (e.g., N2), where it provides high-resolution material decomposition that PET cannot offer. Therefore, integrating quantitative parameters from spectral CT contributes to more precise lymph node staging and target delineation. Nevertheless, given the predominantly retrospective nature and small sample sizes of current evidence, along with the lack of universally validated parameter combinations and cut-off values, prospective validation is urgently needed before these findings can inform nodal staging in routine practice.

The evidence presented above indicates that spectral CT parameters demonstrate clinical feasibility across multiple steps of lung cancer target delineation. However, the ultimate purpose of target delineation is to inform radiotherapy planning—whether the information provided by spectral CT can genuinely optimize radiotherapy plans and yield dosimetric benefits is the core question to be addressed in the following section.

## 4. Role of Spectral CT in Radiotherapy Planning and Validation

The precision of target delineation ultimately needs to be validated within the radiotherapy plan. This section examines how spectral CT information influences radiotherapy planning and evaluation—including dose calculation based on spectral CT parameters, plan optimization, and treatment verification—thereby comprehensively assessing its practical value across the entire lung cancer radiotherapy workflow.

### 4.1. Improving the Accuracy of Electron Density Calculation

The core of dose calculation in treatment planning systems (TPS) is the conversion of CT numbers to electron density (ED). Conventional single-energy CT values are affected by beam-hardening effects, especially in regions with significant density variations like the lungs, which may lead to inaccurate ED conversion and introduce dose calculation errors [33]. Spectral CT-generated electron density (ED) maps can provide ED data directly, bypassing the need for phantom-based conversion curves [33]. Studies have shown that photon radiotherapy dose calculation based on ED maps is feasible, with no significant differences in dosimetric parameters compared to conventional plans [33,34]. Simultaneously, high-keV monoenergetic images from spectral CT can significantly reduce metal artifacts, providing more reliable CT numbers in complex regions such as tumor-lung interfaces, thereby laying a foundation for improved dose calculation accuracy [35]. A study on NSCLC patients found that despite statistical differences in CT numbers for various tissues (tumor, heart, lung, spinal cord) across different reconstruction energies (40–140 keV)—with values decreasing as energy increased—the target volumes delineated from these different energy images and the resulting radiotherapy plans showed no significant differences in target dose homogeneity, conformity, or organ-at-risk doses. This indicates that the various energy images provided by spectral CT are equivalent in radiotherapy planning, while the stable CT values it provides facilitate more accurate physical parameter estimation [35].

### 4.2. Application Potential in Adaptive Radiotherapy (ART)

Radiotherapy for lung cancer is a dynamically evolving process. Over several weeks of treatment, tumors may shrink or deform due to treatment response, and may also undergo morphological and positional changes due to conditions such as atelectasis. Traditional radiotherapy plans are typically based on a single pre-treatment CT scan and struggle to adapt to such dynamic changes. The core goal of adaptive radiotherapy (ART) is precisely to adjust the plan based on actual tumor changes during treatment. In each localization scan, spectral CT not only provides anatomical morphological information but also allows for rapid assessment of changes in tumor blood perfusion and biological characteristics through quantitative parameters (e.g., iodine maps), providing key evidence for biological ART [36]. For example, iodine maps generated by spectral CT can reflect tissue iodine concentration, serving as a surrogate indicator of tumor perfusion with potential for monitoring treatment response [36]. Studies in cervical cancer radiotherapy have confirmed that spectral CT parameters (such as arterial-phase spectral curve slope λ_HU_ and venous-phase normalized iodine concentration, NIC) are independent predictors for distinguishing lymph node properties, demonstrating high diagnostic performance [37], suggesting potential applicability in dynamic monitoring of lung cancer. Additionally, spectral CT can synthesize virtual non-contrast (VNC) images from a single contrast-enhanced scan, reducing radiation dose while ensuring dose calculation accuracy. Studies have preliminarily validated the feasibility of using VNC images for photon dose calculation in tumors at different sites, including bone metastases, with minimal dosimetric differences compared to true non-contrast images [38]. This significantly optimizes the clinical workflow, enabling convenient acquisition of images suitable for precise dose calculation during each adaptive scan. Furthermore, cutting-edge research is exploring the combination of artificial intelligence (AI) with spectral CT to more precisely extract radiotherapy parameters, further enhancing the accuracy and efficiency of ART [39]. Therefore, with its multi-parametric quantitative imaging capability, spectral CT provides robust technical support for achieving truly precise, biology- and morphology-based dynamic adaptive radiotherapy.

Overall, spectral CT demonstrates certain application prospects in radiotherapy planning and validation; however, its clinical dissemination remains constrained by multiple factors. The following section systematically analyzes the challenges and limitations that hinder the widespread application of spectral CT in lung cancer radiotherapy target delineation.

## 5. Practical Challenges and Technical Barriers to Clinical Implementation

Although the preceding sections have demonstrated multiple potential advantages of spectral CT in lung cancer radiotherapy target delineation, the journey from “technically feasible” to “clinically routine” still faces numerous practical barriers. This section systematically reviews the major challenges currently constraining its widespread adoption from technical, operational, and evidentiary perspectives. It is important to note that the obstacles discussed here pertain to spectral CT technology itself and its clinical translation, which are distinct from the methodological limitations of this narrative review (addressed separately in the Limitations section at the end of this article).

### 5.1. Technical Standardization and Reproducibility Issues

A core challenge in the clinical implementation of spectral CT lies in the lack of technical standardization and result reproducibility. Currently, different vendors employ fundamentally distinct hardware architectures and proprietary algorithms to achieve spectral imaging—for example, GE’s rapid kVp switching, Siemens’ dual-source configuration, and Philips’ dual-layer detector design. These technical differences lead to substantial variability in raw data processing and material decomposition strategies, which in turn often yield quantitatively distinct results (e.g., iodine concentration values) for the same tissue when measured across different platforms. Consequently, there is a notable lack of accepted reference standards for key quantitative parameters, severely hindering the advancement of multi-center studies and widespread clinical application [23]. Taking dual-energy CT and photon-counting detector CT as examples, although both can achieve material decomposition and feature quantification, their technical principles and output metrics often vary by device [40]. This disparity makes it difficult to directly translate or compare conclusions drawn from one center or device to others, impeding the broad validation and clinical translation of spectral CT quantitative imaging biomarkers [23].

Furthermore, scan protocols are another important factor affecting the consistency of spectral parameters. Tube voltage settings, contrast agent injection regimens, and acquisition phase selection significantly alter the final spectral images and quantitative data. For instance, while low-keV monoenergetic images improve tissue contrast, their effectiveness is notably affected by scanning parameters and patient body size [23]. To mitigate these variabilities, consensus efforts must first address acquisition-level standardization—including uniform selection of tube voltage, contrast protocols, and acquisition phase timing. Therefore, establishing standardized scan protocols is essential to ensure comparability and reproducibility of results across different studies and devices. Without unified procedures, target delineation parameters based on spectral CT (such as tumor iodine uptake) may deviate due to variations in technical details, affecting the precision of treatment decisions.

Although studies have shown that quantitative image types from spectral CT (such as monoenergetic images and iodine maps) are theoretically unaffected by tube voltage and patient body size, providing the possibility of standardized workflows, realizing this potential still relies on unified equipment calibration and protocol execution [23]. Beyond acquisition, reconstruction algorithms and quantitative calibration represent equally critical layers of standardization. Different vendors employ proprietary reconstruction kernels and material decomposition models that are not directly interchangeable. Establishing cross-vendor calibration phantoms and harmonization protocols—such as those being explored by QIBA—is essential to enable meaningful cross-platform comparisons and multi-center pooled analyses.

Therefore, advancing spectral CT standardization must proceed along three parallel fronts—(i) consensus on acquisition protocols, (ii) harmonization of reconstruction algorithms, and (iii) cross-vendor quantitative calibration using validated phantoms—prerequisites for realizing its full value in precision radiotherapy.

### 5.2. Image Noise and Radiation Dose Considerations

Balancing image noise and radiation dose presents another major challenge in the clinical application of spectral CT. Material decomposition images, particularly low-keV virtual monoenergetic images, although significantly improving tissue contrast and helping reveal subtle blood supply differences in tumors, often come with higher image noise that may affect image quality and the visibility of fine structures (such as micrometastases or vascular invasion) [21]. High noise can reduce the contrast-to-noise ratio, potentially obscuring true pathological features or creating artifacts, thereby threatening the precision of target delineation. To address this challenge, advanced iterative reconstruction algorithms and emerging deep learning-based reconstruction methods are being widely applied to optimize image quality [40]. For instance, studies have utilized unsupervised deep learning models based on multi-dimensional U-Net combined with an improved patch training method to significantly improve image quality, demonstrating excellent performance in reducing spectral CT image noise [41]. These AI-driven approaches can suppress noise while more effectively preserving structural details and spatial resolution. On the other hand, radiation dose is a factor requiring strict trade-offs. Although spectral CT can acquire multi-parametric information in a single contrast-enhanced scan, thereby eliminating the need for an additional non-contrast scan, the radiation dose from its dual-energy acquisition mode itself must be carefully optimized to comply with the ALARA principle of radiation protection [42]. For lung cancer patients requiring multiple follow-up examinations, cumulative dose is a significant concern. Studies have shown that using tin (Sn) filtration combined with iterative reconstruction techniques can significantly reduce CT radiation dose to some extent [43]. For example, in ultra-low-dose lung scans, photon-counting detector CT using ultra-high-resolution mode can leverage the “small pixel effect” to effectively reduce image noise, providing the possibility of significantly lowering radiation dose [44]. However, an inherent trade-off exists between dose reduction and image quality maintenance, as excessive dose reduction may increase noise and cause loss of diagnostic information. Therefore, in the practical application of lung cancer target delineation, personalized scan protocols must be developed based on clinical needs, keeping radiation dose at the lowest reasonable level while ensuring image quality.

### 5.3. Absence of Clinically Validated Thresholds for Radiotherapy

Despite the wealth of quantitative parameters generated by spectral CT (e.g., iodine concentration, effective atomic number, and spectral curve slope), a significant barrier to their integration into radiotherapy is the absence of universally accepted and clinically validated thresholds for treatment decision-making. For example, while it is known that viable tumor exhibits higher iodine uptake than atelectasis, the precise iodine concentration value that defines the boundary between them for GTV delineation remains undefined. Similarly, no standardized thresholds exist to guide dose painting, to determine early treatment response, or to distinguish radiation-induced inflammation from true tumor progression during follow-up. This challenge is further compounded by the dynamic nature of lung cancer during radiotherapy—tumors may shrink, shift, or develop peritumoral edema and inflammation over the treatment course, while post-treatment changes such as radiation fibrosis and pneumonitis frequently mimic residual or recurrent disease on conventional imaging. These temporal changes necessitate longitudinal monitoring capabilities beyond what static morphological assessment can provide. The establishment of such thresholds requires large, prospective, multi-center trials that correlate quantitative spectral parameters with histopathological confirmation or clinical outcomes—a process still in its infancy. Moreover, thresholds derived from diagnostic imaging cannot be directly extrapolated to radiotherapy, where over- or under-delineation directly affects dose distribution and organ-at-risk sparing. This lack of operational reference standards remains a critical impediment to the routine use of spectral CT in biological target definition and adaptive radiotherapy.

### 5.4. Need for Large-Scale, Prospective, Multi-Center Clinical Validation

The current evidence base for spectral CT’s clinical impact in lung cancer radiotherapy is predominantly derived from small-sample, single-center retrospective studies. While these studies are essential for demonstrating technical feasibility and generating hypotheses—including the promising results observed in tumor–atelectasis differentiation—they do not provide the high-level evidence required for changing clinical practice. Moreover, heterogeneity in patient populations, tumor histologies, and treatment regimens across studies complicates the synthesis of generalizable evidence. To establish reproducibility of these differentiation improvements and demonstrate true clinical impact on endpoints such as local progression-free survival and toxicity reduction, large-scale, prospective, multi-center randomized controlled trials are urgently needed. These trials must use harmonized acquisition protocols across different vendor platforms and correlate spectral CT-guided target delineation—specifically assessing whether improved tumor–atelectasis distinction translates into reduced geographic miss and improved local control—with patient outcomes, comparing it directly to conventional CT-guided treatment. Until such evidence is available, spectral CT parameters should be considered adjunctive to conventional imaging rather than standalone decision-making criteria. Furthermore, beyond these clinical and technical barriers, methodological limitations inherent to the current literature—including the predominance of small-sample, single-center retrospective studies and the lack of standardized acquisition and reporting protocols—further constrain the generalizability of existing findings and underscore the urgent need for well-designed prospective multicenter investigations.

These challenges indicate that technical improvements to spectral CT alone are insufficient to fully resolve the complex issues in clinical translation. The integration of multimodal imaging and artificial intelligence may offer new pathways to overcome these limitations—a direction that will be discussed in the following section.

## 6. Integration with Multimodal Imaging and Artificial Intelligence

Information provided by a single imaging modality is inherently incomplete. This section focuses on image fusion strategies integrating spectral CT with PET/CT and MRI, with emphasis on how such multimodal integration can further improve the precision of biological target volume definition in lung cancer, and also explores the auxiliary role of artificial intelligence in this process.

### 6.1. Complementarity and Integration with PET/CT and MRI

Multimodal medical image fusion aims to synthesize complementary information from different imaging techniques to provide a more comprehensive basis for clinical decision-making in radiotherapy target delineation [45]. In the context of lung cancer, the morphological and functional data provided by spectral CT (e.g., iodine density maps and effective atomic number maps) exhibit clear complementarity with the metabolic information from PET/CT and the superior soft-tissue contrast from MRI.

This inherent complementarity is strongly supported by the quantitative evidence accumulated for PET/CT and MRI in target delineation. For instance, studies have consistently demonstrated that PET/CT fusion imaging significantly alters the gross tumor volume (GTV) delineation compared to CT alone, with a mean volumetric change reaching 48 ± 89.7%, primarily due to its superior capability in distinguishing tumor from obstructive pneumonitis or atelectasis [46]. Similarly, in patients with concurrent atelectasis, a study by Kumar et al. demonstrated that while MRI-based GTVs tended to be smaller than those from CT, the difference was not statistically significant. Crucially, the interobserver variability for MRI was found to be as favorable as that for PET/CT and superior to CT alone in this challenging scenario, as indicated by higher Dice similarity coefficients [47]. These findings not only validate the added clinical value of functional and soft-tissue imaging over conventional CT but also establish a quantitative benchmark—achieving PET/CT- and MRI-level precision—for what spectral CT is expected to contribute.

One of the most challenging clinical scenarios in lung cancer target delineation is distinguishing tumor boundaries from surrounding inflammatory atelectasis or obstructive pneumonitis—a problem that frequently compromises the accuracy of GTV definition on conventional CT. Spectral CT-derived iodine density maps offer a functional dimension by reflecting tissue perfusion, as malignant tumors typically exhibit increased iodine uptake due to angiogenesis, whereas atelectatic or inflamed lung tissue shows different perfusion patterns. Similarly, effective atomic number (Z-effective) maps provide material decomposition information that can help differentiate neoplastic tissue from benign inflammatory changes based on their distinct elemental compositions. These spectral parameters thus serve as functional biomarkers that enhance the visual contrast between tumor and surrounding benign tissue, directly addressing a critical clinical bottleneck in lung cancer target delineation.

This complementarity suggests that fusing spectral CT iodine maps with PET images holds promise for achieving more comprehensive biological target volume (BTV) delineation, thereby integrating tumor vascularity/perfusion characteristics with metabolic activity information (e.g., SUV-derived parameters). Such integration enables the concept of BTV, which incorporates both metabolic activity and vascular/perfusion heterogeneity, potentially guiding dose escalation to biologically distinct subregions within the tumor for personalized radiotherapy. Additionally, fusion with MRI—particularly functional MRI—represents another important research direction. MRI’s superior soft-tissue contrast offers distinct advantages in local tumor staging, assessing invasion of adjacent mediastinal structures, and detecting recurrence [48], all of which are crucial for accurate target delineation in lung cancer radiotherapy. Combining spectral CT’s high spatial resolution with MRI’s excellent soft-tissue contrast could further improve target delineation accuracy, especially in cases where tumor boundaries are obscured by adjacent normal structures. At the technical implementation level, multimodal fusion techniques—such as deep learning-based fusion networks—can adaptively integrate features from different modalities, generating fusion images that preserve key information from each source [49]. These advanced fusion approaches provide higher-quality imaging foundations for clinical diagnosis and treatment planning, ultimately contributing to more precise and individualized lung cancer radiotherapy. Nevertheless, it must be emphasized that such multimodal integration remains at an exploratory research stage. Given the additional costs and logistical complexities involved, its application should be reserved for selected cases where single-modality spectral CT still fails to resolve boundary ambiguity, and should only be pursued when its added clinical value can be justified in the specific clinical context. Cost-effectiveness considerations must precede any routine recommendation of such combined approaches.

### 6.2. Application of Artificial Intelligence in Spectral CT Data Analysis

Artificial intelligence (AI), particularly deep learning-based models, has shown promising potential in analyzing spectral CT data for lung cancer radiotherapy. Specifically, AI models can leverage multi-parameter spectral information—such as monoenergetic images, iodine density maps, and effective atomic number maps—to improve the efficiency and objectivity of automated primary lung tumor segmentation, a critical step in target delineation [50]. Beyond target delineation, the broader application of AI in spectral CT lung cancer imaging has also gained traction; for instance, combining spectral CT-derived parameters (such as iodine concentration and spectral curve characteristics) with AI-based radiomic analysis has shown potential in improving the differentiation of benign from malignant pulmonary nodules and in assessing disease progression [51]. Furthermore, when spectral CT functional parameters are fused with metabolic information from PET, AI-based approaches could achieve more precise and automated biological target volume delineation by integrating complementary perfusion and metabolic features.

Quantitative evaluations of these AI models have demonstrated promising performance. For example, the deep learning-based segmentation model for primary lung tumors achieved a Dice similarity coefficient of 0.82–0.89 and a Hausdorff distance of <5 mm in internal validation cohorts [50]. Similarly, a radiomic model integrating spectral CT multi-parametric features for pulmonary nodule characterization reported an area under the curve (AUC) of 0.89–0.94 for distinguishing benign from malignant lesions, indicating robust discriminative capacity [51]. These quantitative metrics provide a more concrete benchmark for assessing the current state of AI performance in this domain and highlight its translational potential for clinical decision support.

Looking forward, the emergence of photon-counting detector CT—which offers superior spatial and spectral resolution—may provide even higher-quality spectral data for AI models, potentially further enhancing segmentation accuracy and analytical performance in lung cancer radiotherapy planning [52,53]. For a systematic discussion of the methodological limitations inherent to the current AI studies and this narrative review—including retrospective designs, small sample sizes, lack of external validation, and heterogeneity of primary literature—please refer to the Limitations subsection in the Conclusions and Future Perspectives section.

The combination of multimodal integration and AI technologies brings new possibilities for lung cancer radiotherapy target delineation. However, translating these technologies into routine clinical practice still faces numerous practical barriers. It is also important to emphasize that, despite promising preliminary results, most AI-based noise reduction and multimodal fusion approaches have been validated primarily in retrospective datasets or computational simulations. Prospective validation in radiotherapy-specific clinical workflows, including assessment of their impact on inter-observer variability, dosimetric plan quality, and patient outcomes, remains an essential prerequisite before these technologies can be adopted for routine clinical use [50,51]. The final section outlines these key translational challenges and provides a perspective on future development directions.

## 7. Future Development Directions and Technological Prospects

Synthesizing the discussions in the preceding sections, the application of spectral CT in lung cancer radiotherapy target delineation has accumulated a certain technical foundation and clinical evidence. However, there remains a gap between “feasible” and “routine application.” This section provides a perspective on future developments in this field from three dimensions: technological evolution, AI empowerment, and clinical translation.

### 7.1. The Revolution Brought by Photon-Counting CT

Photon-counting detector CT (PCD-CT) marks the latest generation of CT technology and is expected to bring disruptive changes to lung cancer imaging and target delineation [54]. Unlike conventional detectors, photon-counting detectors (PCDs) directly measure the energy of each incident photon and classify them according to energy thresholds, simultaneously acquiring high-spatial-resolution images and full-phase spectral data in a single scan. Its core advantages include: absence of electronic noise, improved radiation dose efficiency, enhanced iodine signal—allowing for reduced radiation dose while producing fewer artifacts and improved image quality. Second, PCD-CT can achieve spatial resolution far superior to conventional CT, which is crucial for detecting small lung cancer lesions. Studies have shown that PCD-CT excels in imaging fine anatomical structures such as the inner ear, bone, small vessels, heart, and lungs, and its high spatial resolution offers unprecedented possibilities for identifying subtle morphological changes in early-stage lung cancer [55]. Additionally, its inherent spectral imaging capability provides improved contrast resolution beneficial for soft tissue evaluation and determining lesion margins, offering more distribution information about malignant spread or metastatic dissemination. This holds significant value for more accurately distinguishing tumor lesions from surrounding normal lung parenchyma or inflammatory tissue [56,57]. Although direct clinical studies on lung cancer target delineation are still in early stages, PCD-CT in cardiovascular imaging has been shown to more accurately display coronary plaques and significantly reduce stent artifacts, suggesting broad prospects for precisely defining lung cancer targets adjacent to vessels or bone [58,59,60].

### 7.2. Real-Time Spectral Imaging and Theranostic Integration

Future precision radiotherapy for lung cancer will evolve toward deep integration of image guidance and treatment processes, where spectral imaging technology, particularly photon-counting CT, will play a crucial role. Developing spectral imaging systems integrated with radiotherapy equipment is expected to enable real-time image guidance and target verification during treatment [61]. Such integrated systems may address the issues of target displacement caused by respiratory motion, organ motion, and anatomical changes in conventional fractionated radiotherapy. Beyond motion management, spectral CT’s functional parameters—particularly iodine concentration as a surrogate for tissue perfusion—offer unique advantages for longitudinal monitoring throughout the radiotherapy course. This capability enables more accurate discrimination of viable tumor from treatment-related benign changes (e.g., radiation-induced inflammation, organizing pneumonia, or fibrosis), which is essential for timely treatment adaptation. Integrating the online or near-line imaging capability of spectral CT into the ART workflow can provide contrast and material-specific information beyond that of conventional CT. An online adaptive radiotherapy system incorporating spectral CT will propel lung cancer radiotherapy into a new era of precision. In this framework, spectral CT data acquired before or during treatment can be used for real-time target re-delineation and radiotherapy plan optimization. Virtual non-contrast images and material decomposition maps (such as iodine maps) provided by spectral CT can more clearly distinguish tumor tissue, atelectatic areas, and normal lung tissue, thereby reducing delineation uncertainty [62].

Furthermore, spectral information facilitates more accurate calculation of radiation dose distribution, especially at tissue interfaces. Although spectral CT systems integrated with linear accelerators are still in the research and concept-validation stages, real-time image guidance has demonstrated feasibility in other fields, for example, in cardiac radioablation using X-ray real-time image guidance, and in interventional procedures via cone-beam CT three-dimensional vascular image fusion for real-time puncture navigation [63,64]. These technological developments lay the groundwork for a future integrated spectral CT radiotherapy platform encompassing diagnosis, planning, real-time guidance, and validation. The ultimate goal is to dynamically adjust radiotherapy regimens within each treatment fraction based on the patient’s real-time anatomical and functional status, maximizing tumor control while minimizing damage to surrounding normal tissues, truly achieving individualized, adaptive precision radiotherapy.

### 7.3. Challenges in Clinical Translation and Future Perspectives

Despite the promising potential of spectral CT for improving target delineation in lung cancer radiotherapy, several barriers impede its widespread clinical adoption. A global survey conducted by the International Atomic Energy Agency revealed that although the installation of spectral CT systems is increasing, clinical utilization rates remain low, with key limiting factors including insufficient training, cumbersome operational workflows, increased image interpretation time, and uncertainty regarding clinical value [65]. These challenges are particularly salient for lung cancer radiotherapy, where a lack of unified operational protocols and parameter interpretation standards persists.

Foremost among these barriers is the lack of high-level clinical evidence. Currently, the vast majority of published studies are single-center, retrospective analyses with relatively small sample sizes, which have primarily demonstrated the feasibility of spectral parameters—such as iodine concentration, spectral curve slope, and effective atomic number—in distinguishing lung tumors from adjacent atelectasis and characterizing intratumoral heterogeneity [42]. However, no large-scale, prospective randomized controlled trials have yet been published to verify whether spectral CT-based target delineation translates into tangible improvements in local progression-free survival, overall survival, or pulmonary toxicity reduction specifically in lung cancer patients. Furthermore, the generalizability of existing radiomic and multi-parametric models derived from spectral CT data remains largely untested across different CT vendors, acquisition protocols, and reconstruction algorithms, raising concerns about their reproducibility in multi-institutional settings. This evidence gap represents the single most important obstacle to the routine integration of spectral CT into radiotherapy workflows.

An additional dimension often overlooked is the influence of lung cancer’s natural history on imaging-based target delineation. Lung adenocarcinomas, particularly those presenting as subsolid nodules, demonstrate highly variable growth kinetics—pure ground-glass nodules may remain stable for years before progression, whereas part-solid nodules tend to evolve more rapidly [66]. This temporal heterogeneity has direct implications for target definition: the timing of imaging acquisition relative to the tumor’s natural growth trajectory may influence GTV delineation, and spectral CT’s quantitative parameters could potentially serve as biomarkers to distinguish indolent from aggressive biology, guiding decisions on treatment timing and intensity. Furthermore, the dynamic changes occurring during and after radiotherapy—including tumor shrinkage, peritumoral inflammation, radiation pneumonitis, and fibrosis—require a longitudinal imaging approach that conventional morphological assessment cannot adequately address. Spectral CT’s functional parameters offer a promising solution to these challenges by providing biologically relevant information that complements anatomical imaging.

From a cost-effectiveness perspective, the adoption of spectral CT—particularly with the emergence of photon-counting detector CT, which entails substantial acquisition and maintenance costs—must be justified by demonstrable clinical benefits in the lung cancer setting [42]. Formal health-economic analyses are urgently required to compare the cost-effectiveness of spectral CT against conventional CT and PET/CT in lung cancer radiotherapy. Such analyses must weigh the higher initial capital expenditure and potential scanning costs against potential cost-saving mechanisms that warrant investigation in the lung cancer population, including: reducing local recurrence through improved target delineation accuracy, thereby avoiding costly salvage therapies; enabling more precise treatment response assessment to optimize radiation dose and fractionation, thus reducing unnecessary treatments and associated complications; and potentially complementing PET/CT in resource-limited settings where access to PET/CT is constrained [67]. However, robust health-economic evaluations based on prospective lung cancer-specific data are urgently needed to support reimbursement decisions and guideline recommendations.

Addressing these translational barriers—encompassing clinical evidence gaps, cost-effectiveness uncertainties, and practical implementation challenges—will require multidisciplinary collaboration among radiologists, radiation oncologists, and medical physicists to develop practical guidelines for spectral CT-based target delineation, standardizing the complete workflow from image acquisition and post-processing to clinical interpretation [65]. Concurrently, structured educational programs—incorporating multidisciplinary team teaching models and competency-based training—are essential to equip radiotherapy professionals with the skills needed to proficiently utilize spectral CT in clinical practice [68,69]. Overcoming these interconnected hurdles will be critical to realizing the full potential of spectral CT in advancing precision lung cancer radiotherapy.

## 8. Conclusions

### 8.1. Clinical Place in Current Practice

Spectral CT represents a significant advancement in lung cancer radiotherapy imaging, offering multi-parametric quantitative information—including iodine density maps, monoenergetic images, and effective atomic number maps—that directly addresses the critical challenge of distinguishing tumors from adjacent atelectasis or inflammation. However, given the current lack of cross-vendor standardization, the absence of radiotherapy-specific validated thresholds, and limited prospective outcome data, spectral CT should be positioned as a problem-solving adjunct for selected challenging cases where conventional CT is insufficient, rather than as a routine replacement for standard imaging.

### 8.2. Core Conclusions

In summary, spectral CT enhances target delineation accuracy by providing functional and compositional information beyond conventional CT, yet its clinical adoption depends on overcoming barriers in standardization, evidence generation, and cost-effectiveness validation. For current practice, it serves as a valuable complementary tool under strict quality control; for the future, it holds promise to become an integral component of precision radiotherapy once these challenges are addressed.

## 9. Limitations

Beyond the technical barriers discussed in Section 5, this narrative review has inherent methodological limitations, including potential selection bias inherent to non-systematic literature retrieval and the predominance of small-sample, retrospective studies in the current evidence base, which preclude definitive quantitative conclusions. Despite these limitations, this review provides a balanced synthesis of available evidence, identifying key controversies, research gaps, and future directions in this evolving field.

## 10. Future Perspectives

Looking ahead, the development of spectral CT centers on two major directions: integration with artificial intelligence for automated segmentation and early response assessment, and synergy with next-generation photon-counting CT for superior spatial and contrast resolution. Translating these technological advances into routine practice requires well-designed multicenter prospective trials to demonstrate added clinical benefit, establishment of standardized workflows from acquisition to interpretation, and multidisciplinary collaboration among radiologists, radiation oncologists, and medical physicists. With continued technological optimization and rigorous clinical validation, spectral CT is poised to become an important supportive tool for precision lung cancer radiotherapy in the near future.

### Literature Search Strategy

Databases: PubMed, Web of Science, and Google Scholar.

Time frame: We primarily focused on high-quality literature published within the recent decade (from 2015 to 2025), while also tracing seminal early works that are fundamental to this field;

Search Queries: We used the following keywords: Spectral CT; Lung cancer; Radiotherapy; Target volume delineation.

Inclusion and exclusion criteria: We prioritized studies published in high-impact journals, recent advances within the past three years, and representative evidence-based findings. Meanwhile, we excluded non-English articles and conference reports available only as abstracts.

Number of Publications: We have also included the approximate number of initial records identified (*n* = 128) and the final number of references cited (*n* = 69), explaining our subjective screening process based on thematic relevance and study quality.

## Data Availability

The data presented in this study are available on request from the corresponding author due to institutional and privacy restrictions.

## Supplementary Materials

The following supporting information can be downloaded at: https://www.mdpi.com/article/10.3390/diagnostics16172859/s1, Table S1: Application Value of Spectral CT in the Whole Radiotherapy Workflow for Lung Cancer.
