# Supplementary material for: Application and Development of Spectral CT in Target Delineation for Lung Cancer Radiotherapy
Source: Diagnostics (Basel). 2026 Sep 5;16(17):2859. doi: 10.3390/diagnostics16172859 (PMC13565179; doi:10.3390/diagnostics16172859)
Supplement: Supplementary file 1 [file diagnostics-16-02859-s001.zip › diagnostics-4510759-supplementary.pdf]

Table S1. Application Value of Spectral CT in the Whole Radiotherapy Workflow for Lung Cancer.

Workflow Phase	Imaging Application	Key Parameter(s)	Primary Clinical Value
Target Delineation	Tumor–atelectasis/inflammation differentiation	Iodine concentration (IC), 40-keV CT number	Improves GTV boundary precision and reduces normal tissue irradiation.
	Necrosis/viability mapping for dose painting	Iodine map	Enables biological target definition and focal dose escalation.
	Lymph node metastasis and histotype prediction	Normalized IC (NIC), λ_{HU}	Facilitates individualized CTV and reduces miss/overirradiation risk.
Treatment Planning	Electron density (ED) map generation	Direct CT-to-ED conversion	Enhances dose calculation accuracy in heterogeneous regions.
	Metal artifact suppression	High-keV monoenergetic images	Optimizes imaging quality near metal implants/foreign bodies.
Response Monitoring	Assessing tumor vascular response	Dynamic IC changes	Supports early response prediction and treatment strategy adjustments.
Adaptive Radiotherapy	Performing combined functional-anatomical evaluation	Iodine maps + anatomical images	Provides imaging biomarkers for biological ART, enabling real-time plan adaptation.

Footnotes: GTV, gross tumor volume; CTV, clinical target volume; ART, adaptive radiotherapy; NIC, normalized iodine concentration; ED, electron density; HU, Hounsfield unit. Published evidence supports that spectral CT improves target delineation versus conventional CT, particularly in distinguishing tumor from atelectasis or inflammation.
